# Inconsistencies in self-reported diabetes in a large panel study: the Survey of Health, Ageing and Retirement in Europe (SHARE)

**DOI:** 10.1186/s12874-023-02137-7

**Published:** 2024-01-11

**Authors:** Bernd Kowall, Carolin Girschik, Susanne Stolpe

**Affiliations:** Institute for Medical Informatics, Biometry and Epidemiology, University Hospital Essen, Hufelandstraße 55, 45147 Essen, Germany

**Keywords:** Diabetes mellitus, Longitudinal study, Panel study, Reliability, Self-report

## Abstract

**Background:**

The validity of self-reported chronic conditions has been assessed by comparing them with medical records or register data in several studies. However, the reliability of self-reports of chronic diseases has less often been examined. Our aim was to assess the proportion and determinants of inconsistent self-reports of diabetes in a long panel study.

**Methods:**

SHARE (Survey of Health, Ageing and Retirement in Europe) includes 140,000 persons aged ≥ 50 years from 28 European countries and Israel. We used data from waves 1 to 7 (except wave 3) collected between 2004 and 2017. Diabetes was assessed by self-report. An inconsistent report for diabetes was defined as reporting the condition in one wave, but denying it in at least one later wave. The analysis data set included 13,179 persons who reported diabetes, and answered the question about diabetes in at least one later wave. Log-binomial regression models were fitted to estimate crude and adjusted relative risks (RR) with 95% confidence intervals (CI) for the associations between various exposure variables and inconsistent report of diabetes.

**Results:**

The proportion of persons with inconsistent self-reports of diabetes was 33.0% (95% CI: 32.2%—33.8%). Inconsistencies occurred less often in persons taking antidiabetic drugs (RR = 0.53 (0.53—0.56)), persons with BMI ≥ 35 kg/m^2^ versus BMI < 25 kg/m^2^ (RR = 0.70, (0.64—0.77)), and poor versus excellent subjective health (RR = 0.87 (0.75—1.01)). Inconsistencies occurred more often in older persons (RR = 1.15 (1.12—1.18) per 10 years increase of age), and persons not reporting their age at diabetes onset (RR = 1.38 (1.31—1.45)).

**Conclusion:**

In SHARE, inconsistent self-report of diabetes is frequent. Consistent reports are more likely for persons whose characteristics make diabetes more salient, like intake of antidiabetic medication, obesity, and poor subjective health. However, lack of attention in answering the questions, and poor wording of the items may also play a role.

## Background

Multiwave panel studies are an important information source for the prevalence and incidence of chronic diseases. However, they often rely on self-reports of health conditions. If the accuracy of such self-reports is limited, this may bias estimates of disease prevalence and incidence. Effect measures of associations between various factors and disease prevalence and incidence may also be biased. The validity of self-reported diseases has often been assessed by comparing them with medical records or register data [1–3]. However, the reliability of self-reports of chronic diseases has much less often been examined.

Diabetes mellitus as a chronic disease is in principle not curable. Remission of type 2 diabetes has been shown to be possible after intensive weight management with substantial weight loss and after bariatric surgery [4, 5]. However, for the vast majority of patients, diabetes is still a chronic condition. Thus, participants of a panel study who reported to have diabetes in one wave should give a „yes “ response in all later waves. The consistency of repeated reporting of diabetes has only rarely been examined [6–11]. One such study was done in a highly specific sample of prostate cancer survivors [7], in other studies, the number of included persons with diabetes was rather small (at most 315 in [6, 8, 11]). In all these studies, substantial proportions of participants gave inconsistent reports of diabetes which ranged from 2.9% in the Health and Retirement Study (HRS) to 39.2% in the Norwegian Women and Cancer Study (NOWAC) [8, 9]. However, these earlier studies differed considerably in the number of waves, in time intervals between the waves, the age of the participants, and definitions of inconsistent reporting of diabetes, so that the proportions of respondents giving inconsistent reports are difficult to compare.

This study uses data of the longitudinal Survey of Health, Ageing and Retirement in Europe (SHARE) [12, 13]. This large study allows us to build a very large sample of people with diabetes who have participated in up to seven waves of the panel over a period of about 12 years. We aim to answer the following research questions: first, to estimate the proportions of persons giving inconsistent self-reports of diabetes; second, to examine a wide range of factors which may have an influence on inconsistent reporting of diabetes (sociodemographic factors, drug intake, age at onset of diabetes, number of visits to the doctor, self-rated health, BMI, etc.).

## Methods

### Study population and data analysis set

SHARE is a longitudinal study with 140,000 persons aged 50 years or older from 28 European countries and from Israel [12, 13]. We used data from waves 1, 2, 4, 5, 6 and 7 of SHARE which were collected in 2004/2005/2006 (wave 1), 2006/2007 (wave 2), 2011 (wave 4), 2013 (wave 5), 2015 (wave 6) and 2017 (wave 7) [14–19]. SHARE participants who took part in any previous wave were invited again for later waves. Moreover, refreshment samples were drawn to compensate for the loss of participants. We did not take wave 3 into account because it has a focus on the life history of the participants and, thus, differs from the other waves. Participants are interviewed every two years. The interviews cover a wide range of topics, including demographics, physical and mental health, cognitive function, health care, lifestyle, social support, housing, employment, pensions, household income, and financial transfers. The study rationale and design have been described elsewhere, and further information on SHARE is available online [12, 13]. SHARE data are available free of charge after registration.

The data analysis set includes all participants who said at least in one wave that they had diabetes or had been told by their doctor to have diabetes and who answered the question about diabetes in at least one later wave.

### Variables

To assess diabetes at wave 1, participants were shown a card with 16 diseases, and they were asked: “Has a doctor ever told you that you had any of the conditions on this card? Please tell me the number or numbers of the conditions. … Diabetes or high blood sugar.” At the subsequent waves, participants were shown the card again and asked: “Has a doctor ever told you that you had/Do you currently have any of the conditions on this card? With this we mean that a doctor has told you that you have this condition, and that you are either currently being treated for or bothered by this condition. Please tell me the number or numbers of the conditions.” Diabetes duration was calculated from the answer on the following question: “About how old were you when you were first told by a doctor that you had diabetes or high blood sugar?” To assess diabetes medication, participants were shown a card with diseases, and asked: „Do you currently take drugs at least once a week for problems mentioned on this card? … Drugs for diabetes.”

Heart disease and stroke were assessed with the same questions as for diabetes. Subjective health was assessed with the question „Would you say your health is (excellent / very good / good / fair / poor)? “. BMI was calculated from self-reports of weight and height. International Standard Classification of Education (ISCED) codes were provided in SHARE in the modul gv_isced. Western Europe includes Austria, Germany, the Netherlands, France, Switzerland, Belgium, and Luxembourg; Northern Europe includes Sweden and Denmark; Southern Europe includes Spain, Italy, Greece, Portugal, Croatia, and Israel; Eastern Europe includes Czech Republik, Poland, Hungary, Slovenia, and Estonia. All variables were assessed at the wave where the persons reported to have diabetes for the first time.

### Definition of inconsistencies in self-report of diabetes

An inconsistency in self-report of diabetes is defined as follows: A patient states that he has diabetes or has been told by a doctor to have diabetes in one wave, but says that he has no diabetes in at least one later wave.

### Statistical analysis

Characteristics of participants in the analysis data set are given separately for each wave (means ± standard deviation for continuous variables, proportions for categorical variables). Each person in the analysis data set took part in at least two waves. For categorical variables, the proportions of persons giving inconsistent self-reports of diabetes are estimated with 95% confidence intervals for each category.

Log-binomial regression models were fitted to assess crude and adjusted relative risks with 95% confidence intervals for the association between various exposure variables and inconsistencies in self-report of diabetes (yes/no) as the outcome. An adjustment set was determined for each exposure variable. Adjustment sets can be found under Table 2.

For each wave, we additionally estimated the proportion of persons who said that they did not have diabetes among persons who said that they took diabetes medication. Moreover, we estimated the proportion of persons who said they had diabetes after an inconsistent self-report of diabetes in an earlier wave.

In a secondary analysis, we performed a multiple imputation, using the SAS procedure PROC MI with the statement FCS DISCRIM (fully conditional specification, discriminant function). The number of imputations was 10. We imputed self-report of diabetes at waves 1, 2, 4, 5, 6 and 7, and, additionally, for covariates which may have an influence on inconsistencies in self-report of diabetes (namely age, sex, marital status, country, ISCED, BMI, subjective health, times the doctor was seen, history of stroke, history of myocardial infarction, intake of diabetes drugs). The imputed value for wave 1 was subsequently reset to missing for persons who participated for the first time in wave 2. This was handled in the same way for participants with later baseline waves.

Analyses were performed with SAS (version 9.4; SAS Institute, Cary, North Carolina).

## Results

All together, 139,010 persons participated with the baseline either in wave 1, 2, 4, 5, 6 or 7. From participants of wave 1, 7,116 did not participate in a later wave. From new participants in wave 2, 6,383 did not participate in a later wave (wave 4: 8,275; wave 5: 6,677; wave 6: 4,334; wave 7: 19,032). Thus, 87,193 persons remained who participated at least in one later wave after baseline. Of these, 13,179 fulfilled the condition that they reported to have diabetes in one wave and gave a self-report of diabetes (positive or negative) in at least one further wave. The mean age of these persons increased from 64.7 years in wave 1 to 72.6 years in wave 7 (Table 1). Between 8.5% to 13.2% of the participants described their health as excellent or very good. Between 18.5% and 22.2% reported an earlier heart attack, and between 4.9% to 7.7% reported an earlier stroke.

**Table 1:** Characteristics of study participants in the data analysis set

			**Wave 1**	**Wave 2**	**Wave 4**	**Wave 5**	**Wave 6**	**Wave 7**
N			3793	5077	8064	9527	10,147	9130
Sex	Male		1831 (48.3%)	2406 (47.4%)	3704 (46.4%)	4625 (48.6%)	4831 (47.6%)	4310 (47.2%)
	female		1962 (51.7%)	2671 (52.6%)	4324 (53.6%)	4902 (51.4%)	5316 (52.4%)	4820 (52.8%)
Age (years)			64.7 ± 9.3	67.0 ± 9.3	68.4 ± 9.3	70.0 ± 9.5	71.2 ± 9.3	72.6 ± 9.0
ISCED	Grade 3–6		1431 (38.1%)	2099 (42.1%)	3761 (47.7%)	4681 (50.2%)	4867 (49.0%)	4553 (50.2%)
	Grade 0–2		2329 (61.9%)	2883 (57.9%)	4126 (52.3%)	4651 (49.8%)	5075 (51.0%)	4515 (49.8%)
Partner-ship	Not alone		2748 (72.5%)	3590 (71.5%)	5345 (67.2%)	6290 (66.9%)	6622 (66.0%)	5874 (64.5%)
	alone		1040 (27.5%)	1429 (28.5%)	2604 (32.8%)	3106 (33.1%)	3404 (34.0%)	3239 (35.5%)
Region in Europe	Northern		476 (12.6%)	582 (11.5%)	484 (6.0%)	840 (8.8%)	787 (7.8%)	646 (7.1%)
	Eastern		0	615 (12.1%)	2956 (36.7%)	2586 (27.1%)	2856 (28.2%)	2753 (30.2%)
	Southern		1788 (47.1%)	2190 (43.1%)	1760 (21.8%)	2706 (28.4%)	3633 (35.8%)	3250 (35.6%)
	Western		1529 (40.3%)	1690 (33.3%)	2864 (35.5%)	3395 (35.6%)	2871 (28.3%)	2481 (27.2%)
BMI (kg/m^2^)			28.7 ± 4.9	28.9 ± 5.0	29.5 ± 5.3	29.2 ± 5.3	29.2 ± 5.2	29.0 ± 5.2
Age at diabetes onset	< 30 years		81 (2.1%)	114 (2.3%)	232 (2.9%)	320 (3.4%)	358 (3.5%)	305 (3.3%)
	≥ 30 years		2172 (57.3%)	2872 (56.6%)	5026 (62.3%)	5998 (63.0%)	6458 (63.6%)	5809 (63.6%)
	Not reported		1540 (40.6%)	2091 (41.2%)	2806 (34.8%)	3209 (33.7%)	3331 (32.8%)	3016 (33.0%)
Times of visits to the doctor per year			10.0 ± 13.0	10.1 ± 12.1	9.5 ± 11.1	9.9 ± 11.7	9.7 ± 12.3	10.3 ± 23.1
Report of earlier diabetes		Yes	2253 (59.6%)	3203 (63.3%)	5947 (73.9%)	7349 (77.3%)	8352 (82.4%)	6629 (72.9%)
		No	1529 (40.4%)	1861 (36.7%)	2104 (26.1%)	2158 (22.7%)	1788 (17.6%)	2462 (27.1%)
Intake of diabetes medication		Yes	1853 (49.0%)	2739 (54.0%)	5162 (64.1%)	6588 (69.3%)	7514 (74.1%)	6435 (70.6%)
		No	1929 (51.0%)	2330 (46.0%)	2883 (35.8%)	2907 (30.6%)	2607 (25.7%)	2634 (28.9%)
Subjective health		Excellent	99 (2.6%)	176 (3.5%)	143 (1.8%)	174 (1.8%)	195 (1.9%)	150 (1.7%)
		Very good	400 (10.6%)	463 (9.1%)	575 (7.1%)	726 (7.6%)	704 (6.9%)	623 (6.8%)
		Good	1275 (33.7%)	1696 (33.5%)	2303 (28.6%)	2987 (31.4%)	2948 (29.1%)	2675 (29.3%)
		Fair	1401 (37.0%)	1814 (35.8%)	3168 (39.3%)	3616 (38.0%)	4045 (39.9%)	3574 (39.2%)
		Poor	609 (16.1%)	918 (18.1%)	1864 (23.1%)	2006 (21.1%)	2251 (22.2%)	2071 (22.7%)
Report of earlier heart disease		Yes	699 (18.5%)	986 (19.5%)	1786 (22.2%)	1892 (19.9%)	1989 (19.6%)	1854 (20.4%)
		No	3083 (81.5%)	4078 (80.5%)	6265 (77.8%)	7615 (80.1%)	8151 (80.4%)	7237 (79.6%)
Report of earlier stroke		Yes	184 (4.9%)	278 (5.5%)	578 (7.2%)	710 (7.5%)	715 (7.1%)	705 (7.7%)
		No	3598 (95.1%)	4786 (94.5%)	7473 (92.7%)	8797 (92.5%)	9425 (92.9%)	8386 (92.0%)

Means ± standard deviation, Proportions (N (%)), *ISCED* International Standard Classification of Education

**Fig. 1:**
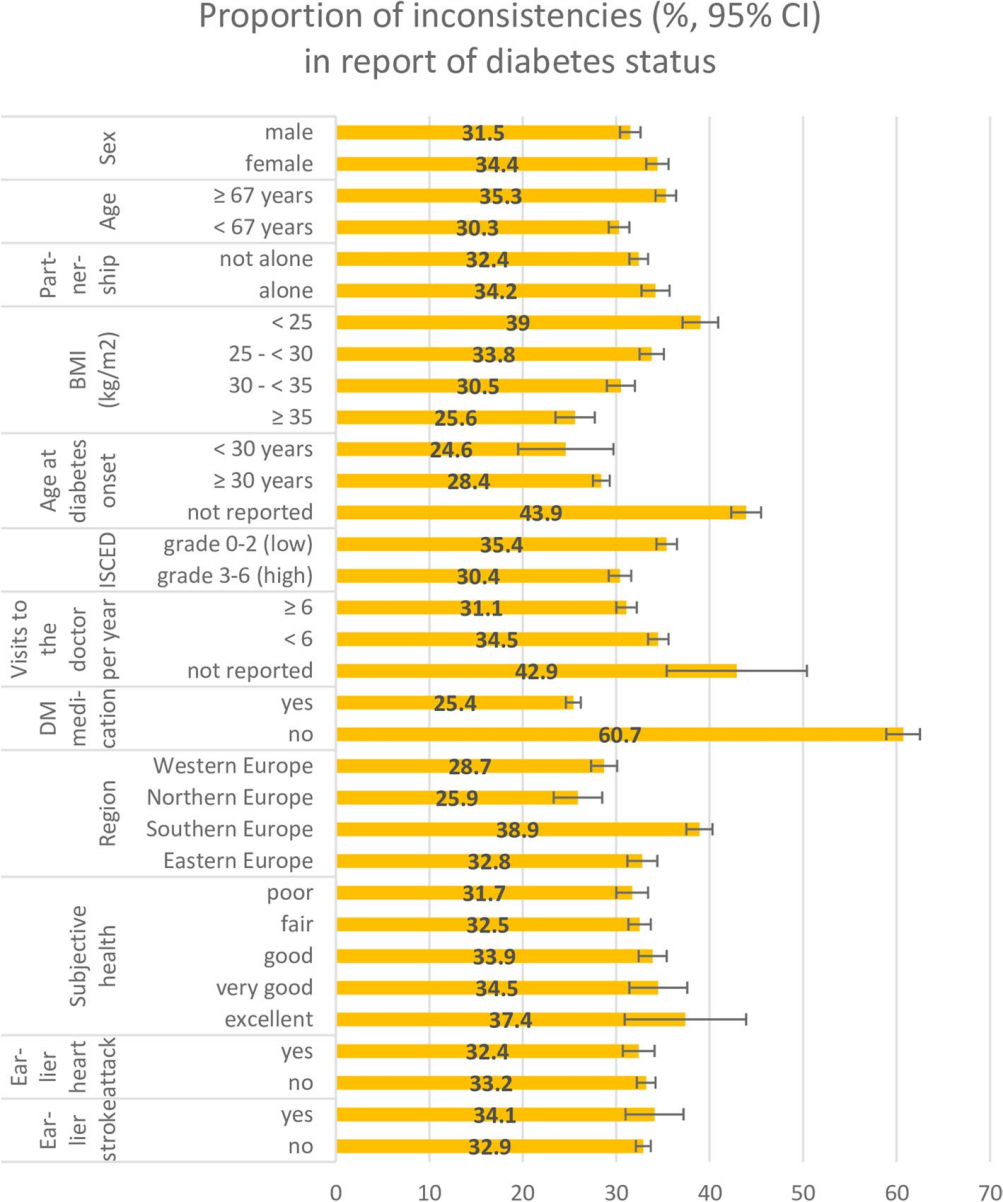
Proportions of inconsistencies in self-reports of diabetes by strata of sociodemographic and diabetes related variables. CI: confidence interval; ISCED: International Standard Classification of Education; DM: diabetes mellitus

The proportion of persons with inconsistent self-reports of diabetes was 33.0% (95% confidence interval: 32.2%–33.8%). This proportion increases with the number of waves participants took part in: for persons participating in 2, 3, 4, 5 and 6 waves, the corresponding proportions of inconsistencies were 24.5%, 31.5%, 36.1%, 43.0%, and 42.0%, respectively. More women than men (34.4 versus 31.5%), more older (≥ the median age, which is 67 years) than younger ones (< 67 years) (35.3 versus 30.3%), and more lower educated persons (ISCED grade 0–2) than higher educated persons (ISCED grade 3–6) (35.4 versus 30.4%) gave inconsistent reports of their diabetes status (Fig. 1). The proportion of persons with inconsistent report of diabetes was lowest in Northern Europe (25.9%) and highest in Southern Europe (38.9%). The proportion of persons with inconsistent diabetes reports decreased from 39.0% in persons with normal weight to 33.8% in persons with overweight to 30.5% in persons with obesity grade 1 and to 25.6% in persons with obesity of at least grade 2. Persons taking no diabetes medication at their first self-report of diabetes were more likely to give inconsistent reports of diabetes at later waves than persons who reported that they took diabetes medication at their first self-report of diabetes (60.7 versus 25.4%). Among persons who did not report their age at diabetes diagnosis, and among persons who did not report how often they saw the doctor per year, the proportion of those giving inconsistent self-reports of diabetes was above average (43.9%, and 42.9%, respectively). The better subjective health was, the higher was the proportion of inconsistent diabetes self-reports (37.4% for excellent, and 31.7% for poor subjective health).

In the adjusted log-binomial regression analyses, women, persons older than the median age, persons from Eastern and Southern Europe, and persons not reporting their age at diabetes onset had a higher risk of giving inconsistent self-reports of diabetes (Table 2). The higher persons´ BMI was, the lower was their risk of inconsistencies in reporting diabetes: persons with a BMI ≥ 35 kg/m^2^ had a relatively 30% lower risk of inconsistencies compared to persons with BMI < 25 kg/m^2^ (RR = 0.70 (0.64–0.77)). Persons describing their health as poor had a relatively 13% lower risk of inconsistencies than persons with excellent subjective health (RR = 0.87 (0.75–1.01)). Persons who reported an earlier heart attack or an earlier stroke barely differed in their risk of giving inconsistent diabetes self-reports from persons without heart attack and stroke. Persons taking diabetes medication at first self-report of diabetes had a lower risk of inconsistencies in diabetes self-report than persons not taking diabetes medication (RR = 0.53 (0.51–0.56)).

**Table 2:** Relative risks for the associations between exposure variables and incidence of inconsistent self-report of diabetes

		***N***	**Number of persons with inconsistent diabetes self-report**	**RR**_**crude**_** (95% CI)**	**RR**_**adj**_** (95% CI)**
Sex	Female	6835	2349	1.09 (1.04 – 1.15)	1.08 (1.03 – 1.13) ^a^
	Male	6344	1999	1	1
Age (per 10 years)		13,179	4348	1.14 (1.11 – 1.16)	1.15 (1.12 – 1.18) ^a^
ISCED	Grade 3–6 (high)	6211	1885	0.86 (0.82 – 0.90)	0.96 (0.91 – 1.02) ^b^
	Grade 0–2 (low)	6828	2416	1	1
Partnership	Not alone	9090	2944	0.95 (0.90 – 1.00)	0.98 (0.93 – 1.03) ^c^
	Alone	3921	1340	1	1
Region	Northern Europe	1010	262	0.91 (0.81 – 1.01)	0.87 (0.77 – 0.97) ^d^
	Eastern Europe	3548	1162	1.14 (1.07 – 1.22)	1.17 (1.10 – 1.25)
	Southern Europe	4431	1723	1.36 (1.28 – 1.44)	1.32 (1.24 – 1.41)
	Western Europe	4190	1201	1	1
BMI (kg/m^2^)	< 25	2432	949	1	1
	25—< 30	5277	1781	0.86 (0.81 – 0.92)	0.88 (0.83 – 0.93) ^e^
	30—< 35	3333	1017	0.78 (0.73 – 0.84)	0.81 (0.75 – 0.87)
	≥ 35	1627	417	0.66 (0.60 – 0.72)	0.70 (0.64 – 0.77)
Age at diabetes onset	< 30 years	260	64	0.87 (0.70 – 1.07)	0.88 (0.71 – 1.08) ^f^
	≥ 30 years	8972	2552	1	1
	Not reported	3947	1732	1.54 (1.47 – 1.63)	1.38 (1.31 – 1.45)
Times of visit to the doctor (per 10 visits)		13,179	4348	0.97 (0.95 – 0.99)	0.97 (0.95 – 0.99) ^g^
Intake of diabetes medication	Yes	10,344	2626	0.42 (0.40 – 0.44)	0.53 (0.51 – 0.56) ^h^
	No	2827	1717	1	1
Subjective health	Excellent	214	80	1	1
	Very good	869	300	0.92 (0.76 – 1.12)	0.93 (0.80 – 1.10) ^i^
	Good	3882	1317	0.91 (0.76 – 1.08)	0.91 (0.79 – 1.06)
	Fair	5271	1715	0.87 (0.73 – 1.04)	0.89 (0.77 – 1.03)
	Poor	2939	932	0.85 (0.71 – 1.02)	0.87 (0.75 – 1.01)
Report of earlier heart attack	Yes	2796	906	0.98 (0.92 – 1.04)	0.97 (0.91 – 1.03) ^j^
	No	10,383	3442	1	1
Report of earlier stroke	Yes	918	313	1.04 (0.94 – 1.14)	1.05 (0.96 – 1.15) ^j^
	No	12,261	4035	1	1

RR_crude_: Crude relative risk; RR_adj _ Adjusted relative risk, *ISCED* International Standard Classification of Education

^a^Adjusted for the number of waves a participant took part in

^b^Adjusted for age, sex, region, number of waves

^c^Adjusted for age, sex, ISCED, region, number of waves

^d^Adjusted for age, sex, ISCED, partnership, number of waves

^e^Adjusted for age, sex, ISCED, region, partnership, number of waves

^f^Adjusted for region, number of waves

^g^Adjusted for age, sex, ISCED, region, partnership, earlier stroke, earlier heart attack, subjective health, number of waves

^h^Adjusted for age, sex, ISCED, region, number of waves, partnership, BMI, subjective health

^i^Adjusted for age, sex, ISCED, region, partnership, earlier stroke, earlier heart attack, diabetes medication, number of waves

^j^Adjusted for age, sex, ISCED, region, partnership, number of waves

In the waves 1, 2, and 4 to 7, between 5.1 to 7.3% of those participants who said that they took diabetes medication said at the same time that they did not have diabetes (data not shown). Among 204 persons who said they had diabetes at wave 1, but not at wave 2, and who participated in at least one of the waves 4 to 7, 108 (52.9% (45.9–60.0)) said at wave 4 or later that they had diabetes. Accordingly, among 643 persons who said they had diabetes at wave 4, but not at wave 5, and who participated in at least one of the waves 6 and 7, 322 (50.1% (46.1–54.0)) said at wave 6 or 7 they had diabetes.

For persons starting in wave 1, the proportions of missing values for self-reported diabetes were 0.3% (wave 1), 10.3% (wave 2), 44.9% (wave 4), 36.6% (wave 5), 40.3% (wave 6), and 47.2% (wave 7) (proportions of missing values for persons starting in later waves not shown). After multiple imputation, the proportion of persons giving at least one inconsistent self-report increased to 39.6% (38.4%—40.8%).

## Discussion

In wave 1 to 7 of SHARE, the proportion of participants who reported diabetes but denied it in at least one later wave was 33%. Participants who were obese, took antidiabetic medication, or reported poor health less often gave inconsistent self-reports of diabetes. In contrast, persons who saw the doctor less often or who did not report their age at diabetes onset more often gave inconsistent reports of their condition. Moreover, inconsistencies appeared more often in women and older persons.

Earlier studies on reliability of self-reports of diabetes in panel studies mainly showed large inconsistencies, too. However, it is difficult to compare results from different studies because studies vary in the number of panel waves, time intervals between the waves, characteristics of study participants, the wording of questions about diabetes, and the definition of inconsistencies. As a matter of course, participants have more opportunities to give inconsistent reports of their condition when they take part in more panel waves – so more persons give inconsistent reports when they participate in more waves as it was also observed in the present study. The exact wording of the question whether a participant had diabetes strongly influences the proportion of inconsistencies. In the Health and Retirement Study (HRS), participants who had reported diabetes in an earlier wave were reminded of this earlier report in later waves [9]. It is sensible to assume that respondents do not want to give inconsistent reports in the presence of the interviewer, and, therefore, in the HRS, the proportion of persons giving inconsistent reports of diabetes was only 2.9%. When algorithms to estimate proportions of inconsistencies differ this also has a strong impact on the study results. For example, in a study on prostate cancer survivors, the denominator also included persons who reported that they had no diabetes in all waves they took part in [7], and inconsistent response patterns for diabetes were given by 7% of the respondents. However, recalculating this figure using the definition of inconsistence applied in the present study led to 33.8% of persons giving inconsistent responses for that study.

In the present study, inconsistent responses were given less often when respondents had characteristics which make their condition more salient. This is the case when they take antidiabetic drugs, are obese, see the doctor more often, or report poor health. Moreover, in this study, inconsistencies were more frequent in persons who did not report the age of diabetes onset. It seems plausible that persons who do not know their age of diabetes onset have less involvement with their disease, and are more likely to give inconsistent self-reports. Inconsistent self-report of diabetes was more frequent in participants with age at diabetes onset ≥ 30 years than in participants with age at onset < 30 years. This seems plausible because older patients with early onset of diabetes are confronted with their health condition for several decades, and should therefore be more aware of it. Persons with age at diabetes onset < 30 years may more often have type 1 diabetes, but age at onset is only a poor proxy to distinguish type 1 and type 2 diabetes. Associations between type of diabetes and inconsistent reporting of diabetes have so far only been investigated in the NOWAC Study showing only small differences between persons with type 1 and type 2, respectively [8]. However, in the NOWAC Study, the distinction between type 1 and type 2 diabetes was based solely on the age at diabetes onset [20]. In the present study, women, and older persons gave more inconsistent reports of diabetes. For age, this result is in line with results from other studies [6, 7, 9]. A decrease in consistent reports with older age may be explained by worsening of memory in older age in general, and, moreover, by an increased risk of cognitive decline in persons with diabetes [21–24]. For sex, results were mixed: in the HRS, and in the Danish Health and Morbidity Study, hardly any association between sex and inconsistent reporting was found, whereas women gave less inconsistent reports in NHANES I [6, 9, 11]. The result in the present study may be made more plausible by the possibility that women who had gestational diabetes during pregnancy may be confused about how to take this into account when answering the question whether a doctor had ever told them they had diabetes. To avoid this, 'outside pregnancy' should be added to the item. Like in the present study, associations with education were generally weak in other studies, and only in NOWAC and in the Taiwanese Survey of Health and Living Status of the Elderly, inconsistent reports of diabetes were given less often by the higher educated [6, 8].

Several reasons for inconsistent self-reports of diabetes are conceivable: (1) Participants with diabetes do not report the disease because they do not have any diabetes related complaints at the time of the interview. (2) Participants may be unwilling to report their health condition for fear of discrimination or out of shame. However, if these potential psychological barriers actually played a role, the question comes up why they had not prevented the same participants from reporting diabetes in an earlier interview. (3) Participants forgot the diagnosis given by their doctor which may be due to cognitive impairment in some older participants. (4) Their doctors may have given them a diagnosis of prediabetes which they misunderstood as a diagnosis of diabetes. In this case, the earlier self-report was wrong when they reported diabetes in one wave but not any more in a later wave. However, positive predictive values of self-reported diabetes are usually assessed as high or very high, and are larger than 90% in many, albeit not in all studies [25–29]. (5) The diagnosis of diabetes given by the doctor was wrong. This is very unlikely because there are clear glucose and HbA1c based definitions of diabetes [30]. (6) Some participants may have lacked attention during the interviews as suggested by 5 – 7% of participants who did not report diabetes although they had reported taking antidiabetic drugs in the same interview.

As estimates of diabetes prevalence and incidence may be biased by inconstencies of diabetes self-report efforts must be taken to reduce the frequencies of these inconsisties. One measure is to asssess not only present diabetes or former diagnoses of diabetes but also the intake of antidiabetic medication and age at onset. Thus, corrections could be made for persons who report the latter but give no report of diabetes. Moreover, participants should be reminded of their earlier statements of diabetes („in the last interview you said that … “) – so participants with an earlier „yes “ response and a later „no “ response may either dispute their earlier „yes “ response or think their later „no “ response over. Furthermore, showing interviewees lists with 16 or more disases as done in SHARE and ask them which health conditions apply to them may be overwhelming for a part of the older participants. Instead, each disease should be addressed separately. In addition, questions about diabetes could be asked a second time. For example, in the Heinz Nixdorf Recall Study, participants are asked about diabetes once more when blood samples are taken [31]. Finally, the question about diabetes in SHARE should be worded more clearly. The additional explanation „ With this we mean that a doctor has told you that you have this condition, and that you are either currently being treated for or bothered by this condition “ might bring participants who do not suffer from diabetes at the time of the interview to give a „no “ response.

This study has advantages over previous studies. With an analysis data set of 13,179 participants with diabetes it is by far the largest study to examine inconsistencies of self-reports of diabetes in panel studies – earlier studies were either much smaller or focussed on a specific population [6–11]. Moreover, the data set included seven waves with time intervals of two years allowing some specific additional analyses (e.g., on correction of inconsistencies in later waves). Additionally, we took a wide range of determinants of inconsistencies into account some of which had not been adressed in earlier studies. This study also has limitations. There are no data from medical records like glucose and HbA1c values which would make it possible to look for false positive and false negative self-reports of diabetes. Unawareness of diabetes is still frequent [32, 33], and blood parameters would help to improve and deepen the analyses of this study. As indicated above, a further limitation of this study was that type of diabetes was not asked.

## Conclusions

Although diabetes is in principle not curable and should be reported again after a „yes “ response in the past, inconsistencies in self-report of diabetes are frequent. Measures should be taken to make this phenomenon less likely in panel studies, in particular by wording questions about diabetes more clearly. Factors like obesity, intake of antidiabetic drugs or poor self-rated health which make diabetes more salient for a patient lead to lower frequencies of inconsistent self-reports of diabetes.

## Data Availability

SHARE data are available free of charge.
